# Neuronal Glycogen Breakdown Mitigates Tauopathy via Pentose Phosphate Pathway-Mediated Oxidative Stress Reduction

**DOI:** 10.21203/rs.3.rs-3526342/v1

**Published:** 2023-11-08

**Authors:** Sudipta Bar, Kenneth A. Wilson, Tyler A.U. Hilsabeck, Sydney Alderfer, Eric B. Dammer, Jordan B Burton, Samah Shah, Anja Holtz, Enrique M. Carrera, Jennifer N. Beck, Jackson H Chen, Grant Kauwe, Tara E. Tracy, Nicholas T. Seyfried, Birgit Schilling, Lisa M. Ellerby, Pankaj Kapahi

**Affiliations:** 1 Buck Institute for Research on Aging, Novato, CA 94947, USA; 2 Department of Biochemistry, Emory University School of Medicine, Atlanta, GA 30322, USA; 3 Emory Center for Neurodegenerative Disease, Emory University School of Medicine, Atlanta, GA 30322, USA; 4 Emory University, School of Medicine Core Labs, Atlanta, GA 30322, USA

## Abstract

Tauopathies encompass a range of neurodegenerative disorders, such as Alzheimer’s disease (AD) and frontotemporal dementia (FTD). Unfortunately, current treatment approaches for tauopathies have yielded limited success, underscoring the pressing need for novel therapeutic strategies. We observed distinct signatures of impaired glycogen metabolism in the *Drosophila* brain of the tauopathy model and the brain of AD patients, indicating a link between tauopathies and glycogen metabolism. We demonstrate that the breakdown of neuronal glycogen by activating glycogen phosphorylase (GlyP) ameliorates the tauopathy phenotypes in flies and induced pluripotent stem cell (iPSC) derived neurons from FTD patients. We observed that glycogen breakdown redirects the glucose flux to the pentose phosphate pathway to alleviate oxidative stress. Our findings uncover a critical role for increased GlyP activity in mediating the neuroprotection benefit of dietary restriction (DR) through the cAMP-mediated protein kinase A (PKA) activation. Our studies identify impaired glycogen metabolism as a key hallmark for tauopathies and offer a promising therapeutic target in tauopathy treatment.

## Main

Tauopathies encompass a group of neurodegenerative conditions characterized by aberrant aggregation of microtubule-associated protein tau (MAPT)^1,2^. Despite identifying hyperphosphorylated neurofibrillary tangles (NFTs) of tau protein in the brain and additional genetic risk factors for tauopathy, therapeutics to treat the disease have proven challenging^3^. Hypometabolic conditions in the brain stemming from altered glucose metabolism have been reported in multiple tauopathy diseases such as Alzheimer’s disease (AD), frontotemporal dementia (FTD), progressive supranuclear palsy (PSP) syndrome, and other related disorder^5–8^. Abnormal glycogen metabolism in neurons is associated with impaired learning and memory formation^9^. The presence of atypical glycogen accumulation in AD, amyotrophic lateral sclerosis (ALS), ischemic stroke, and Lafora disease suggests a potential correlation between abnormal glycogen metabolism and neurodegeneration^10–13^. Glycogen, a stored form of sugar, is an energy source during nutrient-deprived conditions and is predominantly found in the liver and skeletal muscle^14^. The brain contains small amounts of glycogen, mainly stored in astrocytes, where it serves as an energy source for neurons^15,16^. Neurons also contain small amounts of glycogen; however, the specific function of neuron-specific glycogen remains poorly defined^17^.

Dietary restriction (DR) stands out as a highly robust method to extend lifespan and delay the onset of neurodegeneration in yeast, fly, and rodent models of neurodegenerative diseases^18–26^. Nevertheless, it remains imperative to unravel the underlying mechanisms responsible for protecting against neurodegeneration, as this knowledge can significantly enhance our capacity to combat these debilitating conditions. Our study shows that DR significantly ameliorates pathology in tau fly models that overexpress pathogenic human *tau*^*R406W*^ in neurons^27^, demonstrating an intriguing link between tauopathy and dietary restriction. We delineate the underlying mechanisms by which DR confers neuroprotection against tauopathy. We found a significantly higher glycogen accumulation in the brain of *Drosophila,* which DR rescued. Enhancing neuronal glycogen breakdown by overexpressing the enzyme glycogen phosphorylase (GlyP) reversed the tauopathy phenotypes in the *tau*^*R406W*^ fly model and induced pluripotent stem cell (iPSC)-derived neurons from FTD patients. We show that DR promotes glycogen catabolism in the fly brain, highlighting the crucial role of DR in mediating neuroprotection. Our metabolomics and genomics analyses suggest that the breakdown of glycogen in neurons redirected glucose flux towards the pentose phosphate pathway (PPP) instead of glycolysis to mitigate oxidative stress. Furthermore, we demonstrate the regulatory mechanism by which DR activates GlyP by activating cAMP-mediated PKA. Similarly, activating this pathway using 8-Br cAMP also mitigates tau pathology, indicating the potential for therapeutic interventions that break down glycogen to manage tauopathy. Our findings suggest that enhanced neuronal glycogenolysis which is enhanced by DR improves neuronal health by reversing tauopathy phenotypes.

## Results

### DR increases lifespan and protects against neurodegeneration in tau flies.

**Fig 1.**
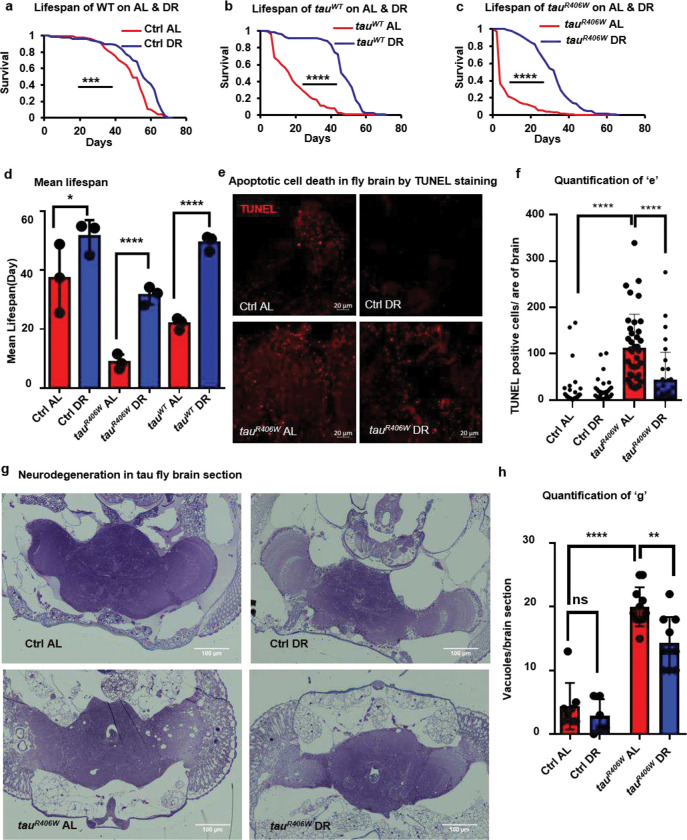
DR increases lifespan and protects against neurodegeneration in tau flies **a**, Lifespan of control (Ctrl) flies, *elav-Gal4*/+ on AL (red) & DR (blue) diets, show extension on DR. **b**, Lifespan of flies expressing *tau*^*WT*^ in the neuron shows extension on DR. **c**, Lifespan of flies expressing *tau*^*R406W*^ in the neurons shows extension on DR. **d**, Mean lifespans of control, *tau*^*WT*^ and *tau*^*R406W*^ flies are increased on DR over flies raised on the AL diet. This experiment represents the mean values of three independent experiments. **e**, TUNEL staining of control & *tau*^*R406W*^ fly’s midbrain on AL & DR. Red dots indicate TUNEL-positive nuclei, which are increased in *tau*^*R406W*^ on AL diets and rescued by DR diet. **f**, Quantification of TUNEL staining show that the number of TUNEL-positive cells per area of the *tau*^*R406W*^ fly brain increases on AL diet and is rescued by DR. Dots represent individual fly brains. See also Figure S1. **g**, Semi-thick sections of *tau*^*R406W*^ fly brain stained with toluidine blue shows increased vacuoles in *tau*^*R406W,*^ which is rescued by DR. **h**, Quantification of vacuoles per brain section showing that DR rescues increased vacuoles in *tau*^*R406W*^. Data in panel D represents 3 independent experiments. An asterisk (*) indicates a significant difference between experimental groups and controls, with the level of significance denoted by the number of asterisks p < 0.05 for *, p < 0.01 for **, p < 0.001 for *** and p < 0.0001 for **** by log-rank test (a, b, and c) or by one-way ANOVA (d, f, and h). Data in bar graphs are presented as mean ± SEM.

We investigated the impact of modulating dietary conditions on *Drosophila* models of tauopathy, where human *tau*^*R406W*^ and *tau*^*WT*^ proteins were overexpressed using the *elav-Gal4* pan-neuronal driver. These tauopathy models exhibited neurodegeneration and reduced lifespan, consistent with previous reports^27^. Flies grown on the *ad libitum* (AL) diet (5% yeast)^28,29^ expressing *tau*^*R406W*^ in the neurons had a mean lifespan of 8.7 days, while those expressing *tau*^*WT*^ had a mean lifespan of 21.8 days compared to 37.2 days for control flies (*elav-Gal4*/+). Mutant tau flies reared on the DR (0.5% yeast) diet showed a statistically significant (log-rank test) 3.5-fold increase in mean lifespan (Fig. 1a–d and **Extended Data Fig. 1a-c**). Additional statistical analysis using the Cox proportional hazard ratio showed a significant interaction between diet and disease for both *tau*^*WT*^ and *tau*^*R406W*^ (**Extended Data Fig. 1d**). Although DR rescued the lifespan of both *tau*^*WT*^ and *tau*^*R406W*^ disease models, its effect was more robust in the *tau*^*R406W*^ model; thus, we primarily used the *tau*^*R406W*^ fly model to understand tauopathy and its interaction with diet. We further investigated the neuroprotective effects of DR in the *tau*^*R406W*^ fly model by utilizing TUNEL staining to assess apoptotic cell death and toluidine blue staining to observe gross morphological changes in the brain. *Tau*^*R406W*^ fly brains showed a significant (p<0.0001) increase in TUNEL-positive cells compared to control flies, which is reduced by 62.6% in DR (Fig. 1e, 1f, and **Extended Data Fig. 1e**). DR also significantly (p<0.005) reduced vacuoles in *tau*^*R406W*^ fly brain tissues compared to flies reared on the AL diet (Fig. 1g and 1h). Overall, these results demonstrate that dietary yeast (the primary source of protein) restriction protects from neurodegeneration and thus improves the lifespan of the tauopathy fly models. Therefore, identifying the mechanisms by which DR confers neuroprotection will elucidate a valuable target for tauopathy.

### Glycogen metabolism is altered in tauopathy, and glycogen breakdown prevents neurodegeneration in *Drosophila* and iPSC-derived neurons.

**Fig. 2:**
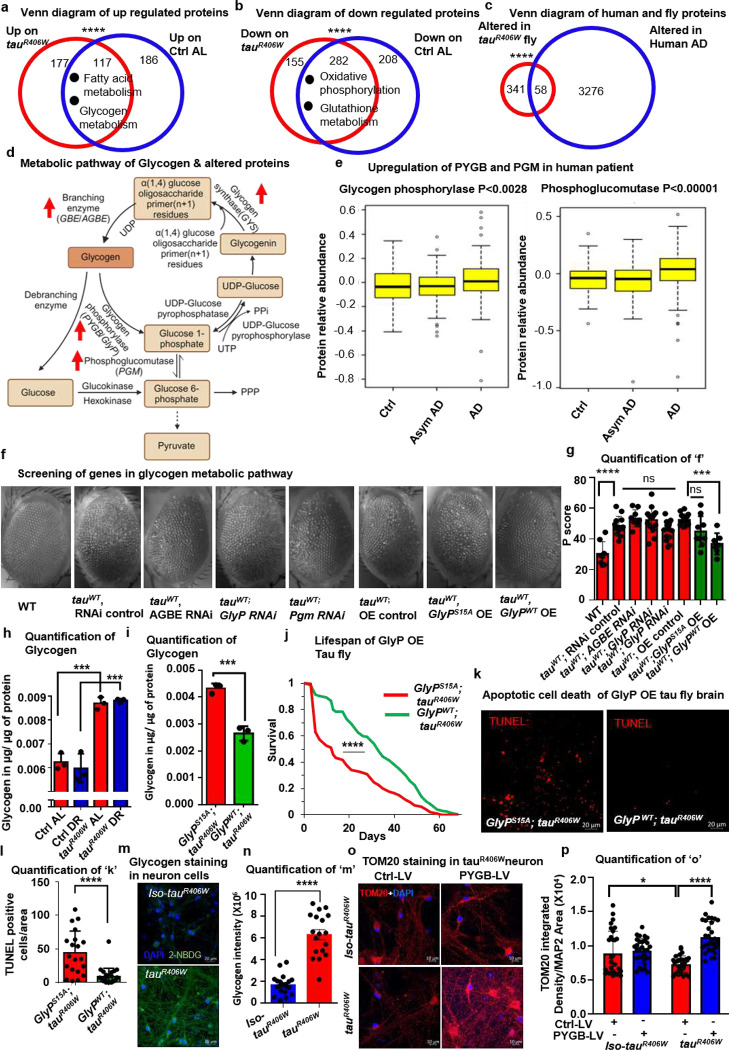
Glycogen metabolism is altered in tauopathy, and glycogen breakdown prevents neurodegeneration in *Drosophila* and iPSC-derived neurons **a**, Venn diagram of the number of proteins upregulated in *tau*^*R406W*^ (red circle) as well as in control on AL diets (blue circle). Dots represent enriched pathways of overlapping proteins, including fatty acid and glycogen metabolism. **b**, Venn diagram shows numbers of proteins down-regulated in *tau*^*R406W*^ (red circle) and control on AL diets (blue circle). Dots represent enriched pathways of overlapping proteins, including oxidative phosphorylation and glutathione metabolism. **c**, Venn diagram of the overlapping genes altered in humans and flies. The red circle represents altered protein with a human orthologue, and the blue circle represents altered protein in a human AD patient’s brain. **d**, Schematic diagram of glycogen metabolism with upregulated proteins marked with red upright arrows. **e**, Correlation of glycogen phosphorylase and phosphoglucomutase protein abundance with AD diagnosis. Asym AD represents asymptomatic AD **f**, Images show eye degeneration by overexpression of *tau*^*WT*^ in the eye driven by GMR, a constitutively active stable regulator rescued by *GlyP*^*WT*^ overexpression. Either RNAi or overexpression construct was activated by *elav Gal4*. **g**, Quantification of the phenotypic score derived from either RNAi or overexpression flies shows GlyP^WT^ overexpression rescues the phenotypic score of eye degeneration. **h**, Quantification of glycogen (in μg/μg of protein) in *tau*^*R406W*^ or control on both AL and DR diets shows increased glycogen in *tau*^*R406W*^ fly brain. The red bars represent flies on the AL diet, and the blue bars represent flies on the DR diet. **i**, Quantification of glycogen (in μg/μg of protein) of control (*tau*^*R406W*^ expressing mutant *GlyP*^*S15A*^) and overexpression of *GlyP*^*WT*^ in *tau*^*R406W*^ fly brains show a reduction in *GlyP*^*WT*^ in *tau*^*R406W*^. **j**, Increased lifespan of *GlyP*^*WT*^*; tau*^*R406W*^ (green) compared to control *GlyP*^*S15A*^*; tau*^*R406W*^ flies(red). **k**, TUNEL staining of whole mount fly brains of *GlyP*^*S15A*^*; tau*^*R406W*^ and *GlyP*^*WT*^*; tau*^*R406W*^. Red dots represent TUNEL-positive cells reduced in the midbrain of *GlyP*^*WT*^*; tau*^*R406W*^ flies. **l**, Quantification of TUNEL staining shows that the number of TUNEL-positive cells per brain area is reduced in *GlyP*^*WT*^*; tau*^*R406W*^. Dots represent individual fly brains. **m**, Images represent glycogen staining with fluorescent 2-NBDG in patient iPSC-derived *tau*^*R406W*^ neurons and isogenic control cells(*iso-tau*^*R406W*^). **n**, Quantification of glycogen as fluorescence intensity shows an increase in *tau*^*R406W*^ neurons (red) compared to isogenic control neurons(blue). **o**, Immunocytochemistry of mitochondria labeled with TOM20 (red) counterstained by DAPI (blue) in *iso-tau*^*R406W*^ and *tau*^*R406W*^ neurons with either control lentiviral transduction or *PYGB* overexpressing lentivirus. **p**, Quantification of mitochondrial density normalized with MAP2 area shows that reduced mitochondria in *tau*^*R406W*^ neurons are rescued by *PYGB* overexpression. Each dot represents an image field from n=3 coverslips per condition for N and P. See also Table 1 in supplementary, Figure S2, supplementary data 1, and supplementary data 2. An asterisk (*) indicates a significant difference between experimental groups and controls, with the level of significance denoted by the number of asterisks p < 0.05 for *, p < 0.01 for **, p < 0.001 for *** and p < 0.0001 for **** by Fisher’s exact (a, b, and c), by one-way ANOVA (e, g, and h), by Two-way ANOVA (p), by Student’s t-test (i, l, and n) or by log-rank test (j). Data in bar graphs are presented as mean ± SEM.

To identify the mechanism of DR-mediated neuroprotection, we conducted an unbiased proteomic analysis of the heads of *tau*^*R406W*^ and control flies on AL and DR diets. The proteomic analysis was performed by comparing the different conditions using a quantitative, label-free workflow, data-independent acquisition (DIA)^30,31^. Overall, we were able to identify and quantify >1,500 proteins that were altered due to diet and disease conditions (**Extended Data 1**). Proteins altered in the heads of mutant tau flies compared to controls significantly overlap with those changed upon AL diet compared to DR (Fig. 2a and 2b), further supporting the interaction between diet and tauopathy. Proteomics analysis identified 294 proteins that were upregulated irrespective of diet changes and solely because of pathogenic *tau*^*R406W*^ protein expression, and 303 proteins were upregulated in control fly brains due to rich diets; among these proteins, 117 are common in both conditions (Fig. 2a). A similar analysis identified that there was an overlap of 282 in down-regulated proteins in *tau*^*R406W*^ and control flies on the rich diet (Fig. 2b). Pathway analysis of the 117 common upregulated proteins revealed that the most significant protein sets were related mainly to metabolism, among which fat and glycogen metabolism were top-ranked (Fig 2a and **Extended Data Table 1**). Similarly, GO term analysis of the common 282 downregulated proteins identified oxidative phosphorylation and glutathione metabolism as the most affected pathways (Fig. 2b and **Extended Data Table 1**). A recent unbiased proteomics study using >2000 human brains and about 400 cerebrospinal fluid samples identified that 3334 proteins were altered in AD patients^6^. Cross-comparison of our *Drosophila* data set with the human dataset identified 58 common orthologues altered in *tau*^*R406W*^ fly brain and human AD patients (Fig. 2c and **Extended Data 2**). The glycogen metabolism-related proteins GlyP, phosphoglucomutase (PGM), glycogen synthase (GyS), and 1,4-alpha-glucan branching enzyme (AGBE) were significantly upregulated in both *tau*^*R406W*^ and on the AL diet (Fig. 2d). Within the human data set, we found that PYGB (human orthologue of brain-specific GlyP) and PGM were significantly upregulated in AD patients’ brains (Fig. 2e).

We screened the glycogen metabolism-related candidate genes by neuronally overexpressing or downregulating these candidates in a fly where *tau*^*WT*^ was stably expressed in the eye using the glass multimer reporter (GMR) regulatory sequence^32,33^. To minimize the potential additive lethal effects of candidate genes, expression of tau^WT^ was restricted solely to the eye. We used RNAi for *AGBE (*human orthologue is glycogen branching enzyme, *GBE*), *Pgm*, *GlyP*, and overexpression for *GlyP*^*WT*^ and a nonfunctional phosphomutant control fly for *GlyP* (*GlyP*^*S15A*^). Among the tested genes, overexpression of *GlyP*^*WT*^, the critical enzyme of glycogen catabolism, was able to rescue the tau-mediated rough eye phenotype significantly (Fig. 2f and 2g). We observed glycogen accumulation in the *tau*^*R406W*^ fly brain increased by 28.3% and 32.2% on AL and DR diets, respectively, compared to control fly brains (Fig. 2h). Overexpression of wild-type *GlyP* in tau fly brains using the *elav-Gal4* driver for *tau*^*R406W*^ (*GlyP*^*WT*^*; tau*^*R406W*^) reduced glycogen storage by 38.8% compared to the control flies (*GlyP*^*S15A*^*; tau*^*R406W*^) (Fig. 2i). Interestingly, *GlyP* overexpression extended the mean lifespan of *tau*^*R406W*^ flies by 69.7% (Fig. 2j). However, no further lifespan extension was observed on the DR diet with overexpression of *GlyP* (**Extended Data Fig. 2c**). We confirmed that tau expression was not altered between the genotypes (**Extended Data Fig. 2a and 2b**). We also observed that the TUNEL-positive apoptotic cells in the *tau*^*R406W*^ background were reduced by 80% with overexpression of wild-type *GlyP* versus its control (Fig. 2k and 2l). Next, we investigated the glycogen accumulation and the role of glycogen phosphorylase in disease phenotype reversal using human patient iPSC-derived neurons with *tau* mutations. For this purpose, we generated uniform iPSC lines for *tau*^*R406W*^, *tau*^*V337M,*^ and respective isogenic controls that securely house a mouse Ngn2 transgene at a specific integration site within the adeno-associated virus safe-harbor (AAVS1) locus using patient-derived iPSC cells and which can be activated with doxycycline^34,35^. Similar to the fly model, we observed that the *tau*^*R406W*^ neurons accumulated a 3.7-fold increase of glycogen, labeled with fluorescence analog of glucose 2-NBDG, versus isogenic control (*iso-tau*^*R406W*^) neurons with the mutation corrected to wild type tau (Fig. 2m and 2n)^36^. Overexpression of *PYGB*, the brain-specific human ortholog of fly *GlyP*, in *tau*^*R406W*^ neurons by a lentiviral-based expression system reduced glycogen accumulation 3.2-fold versus empty vector transduced control cells (**Extended Data Fig. 2d and 2e**). A previous report showed decreased mitochondrial transport in *tau*^*R406W*^ neurons^37^. Here, we observed a significant reduction of mitochondrial abundance in the *tau*^*R406W*^ neurons compared to isogenic controls, which was rescued by *PYGB* overexpression (Fig. 2o and 2p, **Extended Data Fig. 2f, 2g, and 2h**). We confirmed uniform protein expression by immunolabeling myc-tagged *PYGB* (**Extended Data Fig. 2h**). Furthermore, we found similarly increased glycogen accumulation in iPSC-derived neurons carrying a different FTD-associated tau mutation (*tau*^*V377M*^) (**Extended Data Fig. 2i and 2j**). *PYGB* overexpression also reduced the glycogen storage in *tau*^*V337M*^ neurons (Fig. 2k and 2l). Our findings indicate disrupted glycogen metabolism in the brains of Alzheimer’s disease patients and in *in vitro* models of FTD. Additionally, we observed that activating glycogen catabolism through glycogen phosphorylase overexpression successfully rescued disease phenotypes in both *D. melanogaster* and human iPSC-derived neurons.

### Glycogen breakdown shunts glucose to the pentose phosphate pathway and reduces oxidative stress.

During nutrient deprivation, glycogen breakdown supplies energy by producing the end-product pyruvate via glycolysis. Pyruvate is further converted to acetyl CoA – an essential substrate of the citric acid cycle to produce electron donors NADH and FADH that are additionally utilized for ATP production in oxidative phosphorylation^38,39^. The glycogen breakdown product, glucose-6-phosphate, can be shunted to the PPP, generating reactive oxygen species (ROS) scavenger glutathione (GSH)^40^. Active PPP also produces structural sugars like ribulose-5-phosphate, precursors of nucleotide synthesis.

**Fig 3.**
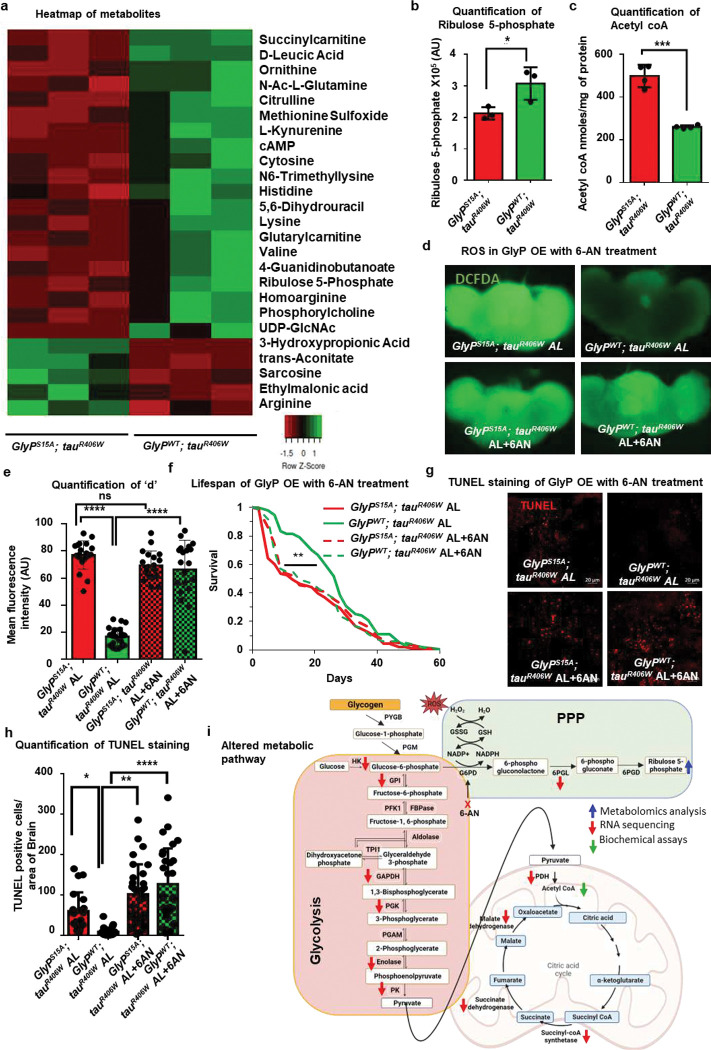
Glycogen breakdown shunts glucose to the pentose phosphate pathway and reduces oxidative stress **a,** Heatmap of significantly (p<0.05) altered metabolites compared between *GlyP*^*S15A*^*; tau*^*R406W*^ and *GlyP*^*WT*^*; tau*^*R406W*^. The green spectrum represents upregulated metabolites, and the red represents downregulated metabolites. **b**, Quantification of Ribulose 5-phosphate shows its abundance is higher in *GlyP*^*S15A*^*; tau*^*R406W*^ (red) than control *GlyP*^*WT*^*; tau*^*R406W*^ (green). **c**, Quantification of acetyl coA shows a reduction in *GlyP*^*S15A*^*; tau*^*R406W*^ (red) fly brain compared to *GlyP*^*WT*^*; tau*^*R406W*^ (green). **d**, Images show ROS staining by DCFDA in the wholemount brains of *GlyP*^*S15A*^*; tau*^*R406W*,^ and *GlyP*^*WT*^*; tau*^*R406W*^ fly with and without 6-AN treatment. **e**, Quantification of fluorescence intensity of DCFDA staining shows a reduction in *GlyP*^*WT*^*; tau*^*R406W*^ (green) compared to *GlyP*^*S15A*^*; tau*^*R406W*^ (red) and increased in 6-AN treatment (green hatched bar). **f**, Lifespan extension of *GlyP*^*WT*^*; tau*^*R406W*^ (green) compared to *GlyP*^*S15A*^*; tau*^*R406W*^ (red) is abrogated with 6-AN treatment (green dashed line). **g**, Images represent TUNEL staining of wholemount brain of *GlyP*^*S15A*^*; tau*^*R406W*^ and *GlyP*^*WT*^*; tau*^*R406W*^ with and without 6-AN treatment. Red dots represent TUNEL-positive cells. **h**, Quantification shows reduced TUNEL positive cells in *GlyP*^*WT*^*; tau*^*R406W*^ (green) than *GlyP*^*S15A*^*; tau*^*R406W*^ (red) and an increase with 6-AN treatment (green checkered bar). **i**, Schematic diagram shows that glycogen catabolism induces the pentose phosphate pathway and reduces the glycolysis and TCA cycle. Arrows represent altered pathway intermediates or enzyme expression. See also Figure S3, Supplementary Data 3, Supplementary Data 4. An asterisk (*) indicates a significant difference between experimental groups and controls, with the level of significance denoted by the number of asterisks p < 0.05 for *, p < 0.01 for **, p < 0.001 for *** and p < 0.0001 for **** by Student’s t-test (b and c), by one-way ANOVA (e and h) or by log-rank test (f). Data in bar graphs are presented as mean ± SEM.

We conducted a targeted metabolomic analysis to identify the metabolic pathways influenced by glycogen breakdown. We identified 25 metabolites significantly altered in *GlyP*^*WT*^*; tau*^*R406W*^ overexpression fly brains versus *GlyP*^*S15A*^*; tau*^*R406W*^ overexpression (**Extended Data 3**). Among these metabolites, 20 were significantly upregulated, and 5 were downregulated (Fig. 3a). Pathway analysis for the altered metabolites identified amino acid metabolism, the urea cycle, and the PPP as the most enriched (**Extended Data Fig. 3a**). Metabolomic analysis showed ribulose 5-phosphate, an essential intermediate of the pentose phosphate pathway, increased by 44.3% in *GlyP*^*WT*^*; tau*^*R406W*^ fly brains (Fig. 3b). Surprisingly, metabolites of the glycolysis or oxidative phosphorylation pathways were not significantly altered. We found that acetyl-CoA - an intermediate between glycolysis and citric acid cycle – was reduced by 47.8% in *GlyP*^*WT*^*; tau*^*R406W*^ flies versus controls (Fig. 3c). Next, we undertook RNA sequencing of *GlyP*^*WT*^*; tau*^*R406W*,^ and its control to determine the changes in metabolic pathways. We identified 473 genes that were significantly downregulated and 546 genes that upregulated in *GlyP*^*WT*^*; tau*^*R406W*^ fly brains (**Extended Data Fig. 4b**). Pathway analysis using significantly altered genes identified oxidative phosphorylation as the most enriched pathway accompanied by the citric acid cycle and glycolysis (**Extended Data Fig. 4c**). We detected a series of glycolytic and citric acid cycle enzymes downregulated in *GlyP*^*WT*^*; tau*^*R406W*^ (Fig. 3i and **Extended Data 4**). Our metabolomics and RNA sequencing results suggest that the breakdown of glycogen does not promote glycolysis.

In the oxidative phase of the PPP, NADPH reduces glutathione (GSH) to scavenge ROS. Next, we tested if the upregulated PPP can reduce ROS in *GlyP*^*WT*^*; tau*^*R406W*^ fly brain. Using DCFDA staining, we observed a 4.5-fold reduction in ROS signal in *GlyP*^*WT*^*; tau*^*R406W*^ fly brains compared to the controls (Fig. 3d and 3e). Additionally, blocking the PPP with 6-amino nicotinamide (6-AN), an inhibitor of glucose 6-phosphate dehydrogenase enzyme, abrogated the rescue effect of *GlyP*^*WT*^ (Fig. 3d and 3e). Treatment with 6-AN also reversed the lifespan extension by *GlyP*^*WT*^. In contrast, control flies noticed no significant lifespan changes (Fig. 3f). In line with these findings, 6-AN abrogated the reduction in apoptotic cell death by *GlyP*^*WT*^ in *tau*^*R406W*^ fly brains (Fig. 3g, 3h, and **Extended Fig. 3d**). Together, our results suggest that *GlyP*-mediated glycogen catabolism downregulates glycolysis but promotes the shunting of metabolites to the PPP, reducing ROS-mediated oxidative stress (Fig. 3i).

### DR activates GlyP by activating the cAMP/PKA pathway.

**Fig. 4:**
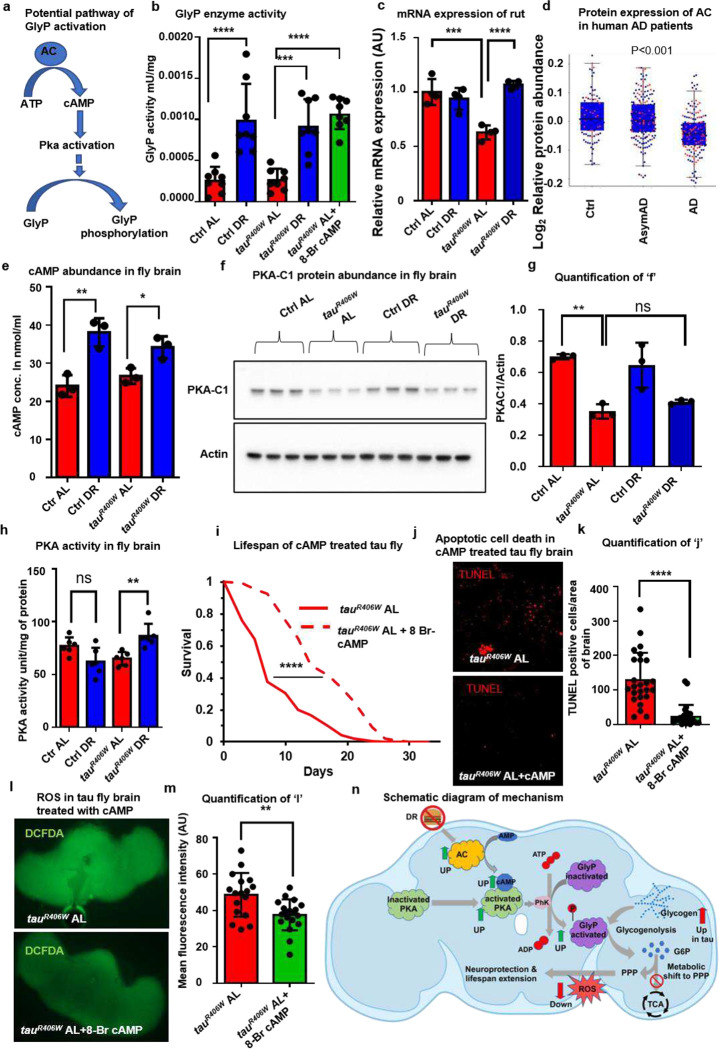
DR activates GlyP by activating the cAMP/PKA pathway **a**, Schematic diagram of upstream activator of GlyP. **b**, GlyP activity in brain lysate of control and *tau*^*R406W*^ on DR and with 8-Br-cAMP treatment on AL diet. Throughout the figure, red, blue, and green colors represent AL, DR, and AL + 8-Br-cAMP. **c**, Relative mRNA expression of the *rut* gene (normalized with RP49) in *tau*^*R406W*^ is downregulated in AL and rescued in the DR diet. **d**, Protein abundance of AC is reduced in AD patients. **e**, cAMP concentration is increased in control and *tau*^*R406W*^ flies on the DR diet compared to the AL diet. **f**, Western blot of PKA-C1 and actin of brain tissue lysate of control and *tau*^*R406W*^ on AL and DR. **g**, Normalized densitometric analysis of western blot shows decreased abundance of PKA-C1 of Tau^R406W^ on AL **h**, PKA activity increases with *tau*^*R406W*^ reared on DR than AL **i**, Lifespan of *tau*^*R406W*^ flies reared on AL is extended with 8-Br-cAMP treatment (dash red). **j**, Images show TUNEL staining of *tau*^*R406W*^ fly brains with and without 8-Br-cAMP treatment. Red dots represent TUNEL-positive cells in the midbrain. **k**, 8-Br-cAMP treatment decreases TUNEL positive cells in *tau*^*R406W*^ flies. Dots represent individual brains. **l**, Images show ROS stained with DCFDA in *tau*^*R406W*^ treated with 8-Br-cAMP. **m**, ROS are reduced in *tau*^*R406W*^ fly brains treated with 8-Br-cAMP. Dots represent individual brains. **n**, Schematic diagram of the mechanism of DR-mediated neuroprotection and lifespan extension by activation of GlyP via cAMP-mediated PKA activation. An asterisk (*) indicates a significant difference between experimental groups and controls, with the level of significance denoted by the number of asterisks p < 0.05 for *, p < 0.01 for **, p < 0.001 for *** and p < 0.0001 for **** by Student’s t-test (k and m), by one-way ANOVA (b, c, d, e, g, and h) or by log-rank test (i). Data in bar graphs are presented as mean ± SEM.

Our proteomics studies identified upregulated GlyP in *tau*^*R406W*^ fly and AD patients’ brains (Fig. 2d and 2e). Upregulation of GlyP could be a cellular response to compensate for the altered metabolism that occurs in response to disease conditions. Under fasting conditions, glycogenolysis could be activated by the cyclic-AMP (cAMP) mediated pathway via activating protein kinase A (PKA) (Fig. 4a)^41^. We aimed to study if DR-mediated neuroprotection and lifespan extension occurred by activation of GlyP. We found that the GlyP enzyme activity was increased 3.5-fold on the DR diet for both *tau*^*R406W*^ and its control (Fig. 4b). Next, we quantified gene expression of adenylate cyclase (*AC*), a potential regulator of GlyP by DR. *Rutabaga* (*Rut*), the *D. melanogaster* orthologue of human AC, showed a significant (p<0.001) decrease in expression in *tau*^*R406W*^ flies fed an AL diet (Fig. 4c). However, dietary restriction (DR) restored the expression levels of *Rutabaga* to those observed in the control group (Fig. 4c). We also found a significant (p<0.001) reduction of AC protein expression in AD patients (Fig. 4d). Furthermore, the DR diet significantly enhanced cAMP concentration in both *tau*^*R406W*^ (p<0.05) and control fly (p<0.01) brains (Fig. 4e), likely due to upregulated AC (Fig. 4c). We noticed that expression of the C1-subunit of PKA (PKA-C1) was significantly reduced in *tau*^*R406W*^ flies head on the AL diet, however, its expression was not altered on the DR diet (Fig. 4f and 4g). It can be posited that enhanced activity rather than an increase in the expression of PKA promotes GlyP function in the DR diet. So, we measured PKA enzyme activity and found that PKA activity increased significantly in *tauR406W* on DR compared to the AL diet (Fig. 4h).

Next, to confirm that the cAMP-mediated pathway activates GlyP, we treated *tau*^*R406W*^ flies with 100μM of 8-Bromo adenosine 3’,5’-cyclic monophosphate (8-Br-cAMP), a hydrolysis-resistant chemical analog of cAMP. We observed that treatment with 8-Br-cAMP rescued the GlyP activity of *tau*^*R406W*^ to the DR level (Fig. 4b). 8-Br-cAMP treatment increased *tau*^*R406W*^ fly lifespan ~2 fold (Figure 4i). No further lifespan extension of *tau*^*R406W*^ was noted on DR supplemented with 8-Br-cAMP (**Extended Data Fig. 4a and 4b**). Treatment with 8-Br-cAMP significantly rescued apoptotic brain cell death (Fig. 4j, 4k, and 4c). 8-Br-cAMP was also significantly reduced ROS in the *tau*^*R406W*^ fly brain (Fig. 4l and 4m). We also observed that *tau*^*R406W*^ flies reared on the AL diet showed a significant (p<0.0001) increase in ROS signal in the brain, which was reduced by 30.74% with the DR diet (**Extended Data Fig. 4d and 4e**). These results demonstrate that DR confers neuroprotection in tauopathy via the cAMP-mediated PKA activation pathway that upregulates GlyP function. Our findings support the notion that DR activates glycogenolysis by enhancing cAMP in neurons, promoting the PPP to reduce ROS and oxidative stress in the brain. Reduced ROS, in turn, protects from apoptotic cell death and thus increases *tau*^*R406W*^ fly lifespan (Fig. 4m).

## Discussion

Several studies have reported that dietary components can pathologically increase tau hyperphosphorylation, a hallmark feature of several neurodegenerative diseases^42–44^. Using a *Drosophila* tauopathy model, we show that dietary protein restriction extends the lifespan and prevents neurodegeneration. Underscoring a key link between tauopathies and DR. Our comprehensive proteomics analysis uncovered a substantial number of proteins exhibiting alterations that coincided with both tauopathy and the protein-rich ad libitum (AL) diet. These identified altered proteins hold promise as potential mediators that shed light on the intricate connection between dietary factors and the development of tauopathy. Our proteomic analysis revealed that the glycogenolytic enzyme GlyP was upregulated in tau fly brains and human AD patients. Notably, we observed that the breakdown of brain-specific glycogen through neuronal overexpression of GlyP reduced neurodegeneration. Overexpression of GlyP in the tau fly brain, and the human brain may be a protective response for survival that reduces tauopathy phenotypes by the breakdown of glycogen.

It has been observed that brain glycogen storage increases in various neurodegenerative diseases, hinting at potential additional functions beyond energy production^11–13^. Our discovery suggests that glycogenolysis in neurons, through the activation of GlyP, directs sugar molecules toward the pentose phosphate pathway rather than activating glycolysis to generate ATP. The oxidative phase of the activated PPP produces reduced glutathione, which acts as a scavenger for reactive oxygen species. Consistent with the role of oxidative stress in tauopathy, we confirmed that GlyP overexpression reduced ROS significantly in the tau fly brain ^45,46^. A recent study revealed that brain glycogen contains a significantly higher amount of glucosamine, at least 25-fold more, compared to glycogen in other organs^47^. Glucosamine is a crucial source of UDP-N-acetylglucosamine, which is involved in N-linked protein glycosylation, a vital cellular process^47^. Our metabolomic analysis identified a significantly higher amount of UDP-N-acetylglucosamine in tau fly heads with *GlyP* overexpression. A study found glucosamine treatment in nematodes reduced glycolysis by 43% and an associated ATP deficit^48^. These findings may explain the mechanism underlying suppressed glycolysis during glycogen breakdown, as observed in our study^47^.

During periods of fasting, cyclic AMP (cAMP) plays a pivotal role in activating glycogenolysis through the activation of PKA and, subsequently, phosphorylase kinase (Phk)^41,49^. Our study elucidates that DR increases *GlyP* activity by cAMP-mediated PKA activation. Treatment with 8-Br-cAMP (cAMP analog) improved lifespan, slowed neurodegeneration, and diminished oxidative stress in the tau fly brain on the AL, but not on the DR diet. This finding confirms that the underlying mechanism of DR overlaps with protection conferred by cAMP activation. Consistent with our findings, it has been shown that administration of rolipram improved cognitive function in an amyloid beta peptide(Aβ)-AD. rat model by inhibiting phosphodiesterase enzyme-4 (*PDE-4*), which converts cAMP to AMP^50^. The report also suggests that the underlying mechanism for this improvement may be attributed to the antioxidant effects of rolipram. Our research revealed that 8-Br-cAMP treatment effectively activates GlyP in the tau fly brain. Therefore, compounds such as PDE-4 inhibitors, including rolipram, have the potential to serve as effective pharmacological agents for GlyP activation, offering a promising strategy for safeguarding against neurodegeneration. In conclusion, our research highlights the importance of glycogen in neurodegenerative disease and the potential of targeting neuronal glycogen breakdown to alleviate oxidative stress, presenting a promising strategy for the management of tauopathies.

## Methods

### Fly strains

*Tau*^*R406W*^ and *tau*^*WT*^ flies were a kind gift by Prof. Mel B Feany27, and the rest of the flies were obtained from Bloomington Stock Center^51^. All strains were outcrossed six times to our lab control *w*^*1118*^ strain. Each line was mated and reared on a standard fly food (1.5% yeast). After 2 days of post eclosion, female progeny were reared on AL (5.0% yeast extract) or DR (0.5% yeast extract) diet^52^. Unless otherwise mentioned, mated flies were grown on AL diets. 8-Br-cAMP and 6-AN treatments were performed by adding 100 μM and 200 μM to AL or DR diets, respectively. All assays were done at the age of 8–10 days. Flies were transferred in new vials every alternative day, and dead flies were documented. The flies were kept in a room with a 12-hour light/dark cycle at a constant temperature of 25°C and a relative humidity of 65%^53^. A comprehensive list of the fly strains utilized in this study can be found at the end of the method section.

### TUNEL staining

The brains of mature *Drosophila* were dissected in PBS and instantly fixed for 30 minutes in 4% paraformaldehyde. TUNEL staining was carried out using the manufacturer’s instructions with some modifications (Roche #11684795910). After fixation, the brains were washed in PBS and permeabilized in 0.3% Triton X-100 and 0.1% sodium citrate. The brains were incubated overnight in TUNEL solution, followed by three washes each for 30 mins. Images were captured using a Zeiss LSM 780 confocal microscope, and quantification was performed by calculating the number of TUNEL-positive cells per unit area of 40X images.

### Toluidine blue staining

Adult fly heads were fixed in 2.5 % glutaraldehyde overnight, followed by post-fixation with 2% osmium tetraoxide for 4 hr. Tissues were then dehydrated with gradually increasing concentrations of ethanol ranging from 30% and followed by 50%, 75%, 95%, and 100%. A final dehydration step was performed with 100% propylene oxide. Each dehydration step was repeated twice for 15 min. Dehydrated tissues were then embedded in epoxy. Semi-thin sections were prepared with a diamond knife and stained with 0.1% toluidine blue.

### DCFDA staining

Fly brains were dissected in S2 media and rinsed twice with PBS before exposure to a 30 μM DCFDA solution in PBS for 10 minutes. The brains were subsequently fixed in 4% paraformaldehyde and washed thrice with PBS. The entire brain mounts were immediately imaged using a Nikon Ni-E upright microscope. Quantification of fluorescence per brain was measured using ImageJ.

### Eye degeneration study

*Drosophila* rough eye phenotype was measured as explained previously using the Flynotyper plug-in in Image J^54^. For this purpose, P{w[+mW.hs]=GawB}elav[C155]; P{w[+mC]=GMR-htau/Ex}1.1 virgin flies were crossed with UAS drive RNAi or overexpression male flies. Progeny flies were used for imaging of the eyes. Images were captured using an Olympus BX51 microscope equipped with a fiber optic gooseneck microscope illuminator and a 10X objective lens. 10–15 optical slices were captured and reconstructed using Zerenestacker (Zerene Systems, Richland, WA).

### Generation of isogenic neurogenein-2 (i^3^N) iPSC-derived neurons

The doxycycline-inducible Neurogenin 2 (Ngn2) transgene was integrated into the AAVS1 locus of human iPSCs using TALENs as previously described^55^. Ngn2 was integrated into Tau^R406W^-carrying human iPSCs and a CRISPR/Cas9-corrected isogenic control line (iso-WTR460W), as well as Tau^V337M^ iPSCs and CRISPR/Cas9-corrected isogenic control iPSCs (iso-WTV337M)^56^. Genomic DNA was extracted from iPSCs with stably integrated Ngn2 using DNeasy Blood and Tissue Kit (Qiagen), and PCR amplification was performed to confirm the presence of a single copy of Ngn2 transgene using PCR3 primers (Forward: CGG TTA ATG TGG CTC TGG TT; Reverse: AGG ATC CTC TCT GGC TCC AT)^35^. Pre-differentiated iPSCs were seeded on 12-mm glass coverslips coated with poly-D-lysine (20 μg/ml; Sigma, P6407) and laminin (0.25 μg/ml; Sigma, L2020) in 24-well plates at a density of 150,000 cells per well.

### Fluorescent Glycogen Detection with 2-NBDG in Human iPSC-Derived Neurons

On day 9, cells were transduced with lentiviral particles expressing human brain-type glycogen phosphorylase (Origene, RC202077L3V) at a multiplicity of infection (moi) of 2. Lentiviral control particles containing the same vector but lacking the glycogen phosphorylase transcript (Origene, PS100092V) were used as a negative control. After 4–5 weeks of maturation, neurons were incubated at 37°C with 500 μM 2-NBDG (APExBIO, B6035) for 4 hours. After incubation, cells were washed three times with PBS. Phenol-red free Neurobasal A (Thermo Fisher, 12348017) was added, and neurons were immediately imaged using a Zeiss LSM780 laser scanning confocal microscope.

### Mitochondria Assay in Human iPSC-Derived Neurons

For immunocytochemistry analysis of mitochondria, neurons were cultured for 30 days and fixed for 15 minutes with 4% paraformaldehyde in PBS. Cells were washed 3 times with PBS followed by 1 hr incubation at RT in blocking buffer (0.1% Triton-X-100, 2% normal donkey serum in PBS). Primary antibodies (rabbit monoclonal IgG to TOM20, 1:400; chicken monoclonal IgG to MAP2, 1:1000) were diluted in blocking buffer and incubated overnight at 4°C followed by 3 washes with PBST (0.1% Triton-X-100 in PBS). Secondary fluorescent-labeled antibodies (donkey anti-rabbit Alexa 555 and donkey anti-chicken Alexa 647, 1:500 each) were added for 1 hr at RT, removed by 3 washes with PBS, and coverslips were mounted onto glass slides with Prolong Gold Antifade with DAPI (ThermoFisher). Confocal images of the neurons were taken using a Zeiss LSM980 63x immersion oil objective, and mitochondria were quantified by TOM20 area normalized to total dendrite density using MAP2 immunolabeled area.

### Biochemical assays

Glycogen measurement was done using a kit protocol (abcam#ab65620). For glycogen measurement, 25 fly heads for each replicate were lysed, and the assay was performed using manufacturer instructions. Background reading from glucose contamination was subtracted according to manufacturer instruction. Glycogen phosphorylase enzyme activity was done using kits (abcam#273271) and following manufacturer instructions with 80 fly heads for each replicate. No enzyme and glycogen were used for background control (abcam#ab273271). cAMP was measured using cAMP ELISA kits (Genscript#L00460) with 80 fly heads for each replicate. Acetyl-Co A was assayed using a kit (Sigma-Aldrich # MAK039) and manufacturer instructions with 30 fly heads for each replicate. Deproteinized tissue lysate was used for the assay. Deproteinization was performed using a kit from Abcam (#ab204708). PKA activity was measured using a kit protocol (ThermoFischer# EIAPKA). For PKA activity, 80 fly heads were used for each biological replicate. The Molecular device’s microplate reader was used for fluorescence intensity and absorbance measurement.

### Proteomics analysis

Proteomics analysis was done in-house. An unbiased proteomics technology to assess differential protein expression using label-free quantification (data-independent acquisitions; DIA), which allowed for comprehensive sampling in a highly quantitative and unbiased fashion, was used^57–59^. 10 days old 25 fly brains were used for each replicate, and 4 replicates were used for each group. The detailed method of proteomics is in the supplementary section.

### Metabolomic analysis

Metabolomics analysis was performed at Northwest Metabolomic Research Center (Seattle, Washington). Metabolites were extracted from 30 fly heads for each group using the protein precipitation method described previously^60,61^. The samples were then homogenized in purified deionized water and mixed with cold methanol containing internal standards (124 μM 6C13-glucose and 25.9 μM 2C13-glutamate). After being stored at −20°C for 30 minutes, followed by sonication and centrifugation, the resulting supernatants were collected, dried, and reconstituted in an LC-matching solvent with additional internal standards (17.8 μM 2C13-tyrosine and 39.2 3C13-lactate). The samples were then transferred to LC vials and analyzed using a temperature-controlled autosampler.

The targeted LC-MS metabolite analysis was conducted on a duplex-LC-MS system consisting of two Shimadzu UPLC pumps, a CTC Analytics PAL HTC-xt temperature-controlled auto-sampler, and an AB Sciex 6500+ Triple Quadrupole MS with an ESI ionization source^61^. The UPLC pumps were connected to the auto-sampler in parallel and performed two independent chromatography separations. Each sample was injected twice onto two identical analytical columns (Waters XBridge BEH Amide XP) in hydrophilic interaction liquid chromatography (HILIC) mode. While one column performed separation and MS data acquisition in ESI+ ionization mode, the other column was equilibrated for sample injection, chromatography separation, and MS data acquisition in ESI− mode. The LC-MS system was controlled using AB Sciex Analyst 1.6.3 software, and MS peaks were integrated using AB Sciex MultiQuant 3.0.3 software. The assay targeted 361 metabolites, including 4 spiked reference internal standards. Across the study set, up to 168 metabolites (plus 4 spiked standards) were measured, and over 90% of the measured metabolites were present in all samples. In addition to the study samples, two sets of quality control (QC) samples were used to monitor assay performance and data reproducibility. QC (I) was a pooled human serum sample used to monitor system performance, and QC (S) was pooled study samples used to monitor data reproducibility. Each QC sample was injected for every 10 study samples. The data were highly reproducible, with a median coefficient of variation (CV) of 4.8%.

### Gene expression analyses

To determine gene expression, RNA sequencing was done at the Novogene facility. The RNA samples were assessed for integrity using the Bioanalyzer 2100 system with the RNA Nano 6000 Assay Kit. Total RNA was used for the sample preparation, wherein mRNA was purified with poly-T oligo-attached magnetic beads. Fragmentation was carried out using divalent cations under elevated temperature in First Strand Synthesis Reaction Buffer(5X), followed by first strand cDNA synthesis using random hexamer primer and M-MuLV Reverse Transcriptase (RNase H−). Subsequently, second-strand cDNA synthesis was performed with DNA Polymerase I and RNase H, and the remaining overhangs were converted to blunt ends via exonuclease/polymerase activities. After the adenylation of 3’ ends of DNA fragments, an adaptor with a hairpin loop structure was ligated for hybridization. Library fragments were purified to select cDNA fragments of preferentially 370~420 bp in length using the AMPure XP system. PCR was performed with Phusion High-Fidelity DNA polymerase, Universal PCR primers, and Index (X) Primer, and the resulting PCR products were purified again using the AMPure XP system. Library quality was assessed on the Agilent Bioanalyzer 2100 system. Following the manufacturer’s instructions, the index-coded samples were clustered on a cBot Cluster Generation System using TruSeq PE Cluster Kit v3-cBot-HS (Illumina). After cluster generation, the library preparations were sequenced on an Illumina Novaseq platform, generating 150 bp paired-end reads.

### Bioinformatics

Gene Ontology (GO) enrichment analysis of proteomics data sets was performed using the String database. Enrichment analysis of metabolomic pathway was performed using MetaboAnalyst server^62^. The heatmap of metabolites was generated using the online tool heatmapper^63^.

### Quantification and statistical analysis

The error bars in the figures indicate the standard error of the mean (SEM) based on a minimum of three biological replicates. An asterisk (*) indicates a significant difference between experimental groups and controls, with the level of significance denoted by the number of asterisks (p < 0.05 for *, p < 0.01 for **, p < 0.001 for *** and p < 0.0001 for ****). These differences were determined using an unpaired t-test or ANOVA with Tukey’s post-hoc test. The statistical significance of the Venn diagrams in Figures 2A, B, and 2C was calculated using Fisher’s Exact Test. All statistical analyses were conducted using GraphPad Prism.

### Cox proportional hazard ratio

We have used Cox proportional hazards analysis implemented in the R package ‘survival’ to analyze the significance of the interaction between two variables in several survival outcomes. We report the probability that B1,2=0, from fitting the formula phenotype=B1*variable1+B2*variable2+B1,2*(variable1*variable2). The respective p values are included in **Extended Data Fig 1d**. Variable 1 is expression of the transgene in the neurons (*Elav*) (No = 0, Yes = 1), and variable2 is either the *tau*^*R406W*^ genotype or *tau*^*WT*^ genotype (without = 0, with = 1), with variable1*variable2 being the interaction term of neuronal expression and tau genotypes.

## Figures and Tables

**Table:** 

Drosophila melanogaster strains used for this study		
Strain	Source	ID number
hTau^WT^ strain, UAS hTau^WT^/TM3.Sb	Provided by the lab of Mel. B. Feany, Department of Pathology, Division of Neuropathology, Harvard Medical School, Boston, USA.	NA.
hTau^R406W^ strain, UAS hTau^R406W^/TM3.Sb	Provided by the lab of Mel. B. Feany, Department of Pathology, Division of Neuropathology, Harvard Medical School, Boston, USA.	NA
Elav-Gal4 Driver (non-inducible, neuronal driver) P{w[+mW.hs]=GawB}elav[C155]	Bloomington Drosophila Stock Center	#458
GMR-driven mutant tau (non-inducible, eye), Elav-Gal4 (non-inducible, neuronal driver) P{w[+mW.hs]=GawB}elav[C155]; P{w[+mC]=GMR-htau/Ex}1.1	Bloomington Drosophila Stock Center	#51360
Transgenic RNAi Project (TRiP) control strain y[1]sc[*]v[1];P{y[+t7.7]v[+t1.8]=VALIUM20-mCherry}attP2	Bloomington Drosophila Stock Center	#35785
Overexpression control strain w[1118];P{w[+mC]=UAS-mito-HA.GFP. AP}2/CyO	Bloomington Drosophila Stock Center	#8442
AGBE RNAi strain, y[1]sc[*]v[1] sev[21]; P{y[+t7.7] v[+t1.8]=TRiP.GL00708}attP2	Bloomington Drosophila Stock Center	#42753
GlyP RNAi strain, y[1]v[1];P{y[+t7.7]v[+t1.8]=TRiP.HMS00032}attP2	Bloomington Drosophila Stock Center	#33634
Pgm RNAi, y[1]sc[*]v[1];P{y[+t7.7]v[+t1.8]=Trip. HMS01333}attP2/TM3 Sb[1]	Bloomington Drosophila Stock Center	#34345
GlyP^WT^ overexpression strain, w[*]; P{y[+t7.7] w[+mC]=UAS-GlyP.P}attP40	Bloomington Drosophila Stock Center	#79211
GlyP^S15A^ overexpression strain, w[*]; P{y[+t7.7] w[+mC]=UAS-GlyP.S15A}attP40/CyO	Bloomington Drosophila Stock Center	#79212

**Table:** 

Cell lines and lentiviral vectors used		
Line	Source	ID
*Tau^R406W^*	Tracy lab, Buck Institute for Research on Aging, CA	NA
*TauM^VSS7M^*	Tracy lab, Buck Institute for Research on Aging, CA	NA
PYGB-myc-DDK	Origene	RC202077L3V
Lenti control virus (pLenti-C-Myc-DDK-P2A-Puro)	Origene	PS100092V

**Table:** 

Cell and fly media and additives used		
mTeSRI Medium	StemCell Technologies	Cat#85850
Matrigel	Corning	Cat#354234
Rock Inhibitor (Y-27632)	StemCell Technologies	Cat#72304
Poly-D-lysine	Sigma-Aldrich	Cat#P6407
Mouse laminin protein	Sigma-Aldrich	Cat#L2020
Doxycycline	Sigma-Aldrich	Cat#D9891
Knockout DMEM/F12 Medium	Thermo Fisher Scientific	Cat#12660012
N2 Supplement	Thermo Fisher Scientific	Cat#17502001
MEM Non-Essential Amino Acids Solution	Thermo Fisher Scientific	Cat#11140050
Brain-derived neurotrophic factor (BDNF)	StemCell Technologies	Cat#78005
Neurotrophin-3 (NT3)	StemCell Technologies	Cat#78074
Neurobasal-A Medium	Thermo Fisher Scientific	Cat#10888022
B-27 Supplement	Thermo Fisher Scientific	Cat#17504044
GlutaMAX Supplement	Thermo Fisher Scientific	Cat#35050061
Nutri-Fly Drosophila Agar, Gelidium	Genesee Scientific	Cat#66-104
Yellow Cornmeal	Genesee Scientific	Cat#62-100
Pure Cane Granulated Sugar	C&H	N/A
Bacto yeast extract	VWR	Cat#90000-722
Saf instant yeast	Rainy Day Foods	

**Table:** 

Antibodies and Stains		
Anti human tau(HT7)	Invitrogen	Cat#MN1000
β-actin rabbit	Cell Signaling Technology	Cat#4967
Pka-C1 polyclonal rabbit	epigenetic	Cat#A64270
Rabbit anti-TOM20	Cell Signaling Technology	Cat#42406
Chicken anti-MAP2	abcam	Cat# ab5392
Mouse anti myc tag antibody(9E10)	abcam	Cat#ab223894
Anti mouse IgG, HRP	Cell Signaling Technology	Cat#7076S
Anti rabbit IgG, HRP	Cell Signaling Technology	Cat#7074S
Donkey anti-mouse IgG Alexa Fluor 488	Thermo Fisher Scientific	Cat#A-21202
Donkey anti-rabbit IgG Alexa Fluor 555	Thermo Fisher Scientific	Cat#A-31572
Donkey anti-chicken IgGAlexa Fluor 657	Thermo Fisher Scientific	Cat#A-78952
Hoechst	AAT Bioquest	Cat#17535
2-NBDG	ApexBio	Cat#B6035
Toluidinblue	Sigma-Aldrich	Cat#198161
DCFDA	Sigma-Aldrich	Cat#D6883

## Data Availability

Raw data and complete MS data sets have been uploaded to the MassIVE repository of the Center for Computational Mass Spectrometry at UCSD and can be downloaded using the following link: doi:10.25345/C54T6FD40 with the MassIVE ID MSV000092637; it is also available at ProteomeXchange with the ID PXD044485. [Note to the reviewers: To access the data repository MassIVE (UCSD) for MS data, please use: Username: MSV000092637_reviewer; Password: winter].
